# Two Famous Doctors

**Published:** 1889-07-27

**Authors:** 


### The Hospital

A Weekly Institutional Journal of

**Science, Medicine, Nursing, and Philanthropy.**

*For Hospitals, Asylums, Medical Practitioners, Students, Nurses, Families, Parishes, Congregations, and the Charitable Public.*

Vol. VI. No. 148.] [July 27, 1889.

### Two Famous Doctors.

Doctors and their doings have been much more interesting to the great world the last two decades than they were during the two hundred years after Bacon had done his best to put an extinguisher on them by declaring that "these sciences (the medical) stand at a stay and have done for many ages." "These sciences" do not "stand at a stay" now; on the contrary, for many people they move both too fast and too far, and occupy altogether more of the public attention than is considered quite satisfactory. To vast numbers the doctor is a decidedly peculiar person, who lives, moves, and has his being in a world shut off and apart from the common toils, common passions, and common experiences of mankind. There is undoubtedly a basis of fact in this, and the doctor himself suffers a certain limiting and diminution of his manhood on that account. It is gratifying to pride to hold any claim to exclusiveness, whether that claim be of birth, of wealth, of distinguished ability and success, or of the possession of knowledge which the many can never hope to gain. But however gratifying it may be to that common but unworthy sentiment, it does not make up for the want of a stronger development on the noblest of all lines, the lines of an unlimited humanity and of a perfect brotherhood. Science declares in the abstract the unity of man and his descent from a common stock; but in practice she no more recognises the claims of the common parentage and relationship than does birth, wealth, or other more familiar kinds of eminence.

At the present moment the medical profession has in England two equal and co-ordinate heads, the President of the Royal College of Physicians, and the President of the Royal College of Surgeons. No doubt, if there is a question of precedence, the College of Physicians will stand first, but for all practical purposes the two colleges and their respective heads stand side by side. Each of them has for its king to-day, perhaps as typical a representative of modern medicine and surgery as could well be found. Sir Andrew Clark, the head of his college, is a physician who has attained to the dignity of a baronetcy. Mr. Jonathan Hutchinson, the head of his college, is a surgeon—the greatest "general practitioner in Europe," the envious call him—who is a Fellow of the Royal Society though not yet either a baronet or even a knight. These two, with all their professional claims to distinction, all their peculiarities as men and citizens, all the work they have done for themselves and their own advancement, and all they have done for their profession and the world, may well be taken as typical representatives and exponents of the two branches of the healing art. As is Sir Andrew Clark, such is the average physician of the times; and as is Mr. Jonathan Hutchinson, such is the average surgeon. He who knows and understands these two, knows and understands the medical practitioners and the medical practice of to-day.

Sir Andrew Clark came to London about thirty-six years ago, a young and unknown Scotchman. Those who know him now can well imagine the general working of his mind at that time. He knew that London was the centre of the British Empire, and the fittest arena for a man who believed he possessed powers which would sooner or later carry him to the front among his fellows. He soon learnt that medical London does not look kindly on youthful aspirants from the country, especially if "the country" happens also to be Scotland. When he obtained his first appointment of importance, that of assistant physician to the London Hospital, some of the disappointed ones are reported to have said, "Let him have it, poor Scotch beggar, he wont hold it long." For Dr. Andrew Clark was in ill-health; and at that time, we believe, was apprehensive of an early death. But he had got what he wanted—a start; and to such men as he a start is generally all that is required. Of his determination of character, of his Scotch wariness, of his power of work, of his skill in carrying his wares to the best market, no one who knows him well entertains the least doubt. Sir Andrew Clark was made for wordly success, and it would have been a miracle if he had not won it. As a physician he is undoubtedly one of the ablest, most painstaking and most anxious to help his patients whom it is possible to consult. What is to be known he strives always to know, what is to be done he desires always to do; and to every separate patient he devotes his mind and energies as thoroughly as if fame and fortune were yet to be acquired.

Such is Sir Andrew Clark the physician. What is Sir Andrew Clark the man? What has he done as a man? What does this age and this nation owe to him scientifically, politically, morally? What has he, as a leading scientist, done for that which Spencer, Huxley, and others of their school, would call the "development of the race?" The question is important, as a similar question would be important, if asked concerning the Lord Chancellor, the Archbishop of Canterbury, the Prime Minister, or any other conspicuous figure of the period. In his consulting-room and his hospital, Sir Andrew Clark has achieved high distinction. In the great world, and for the great world has he accomplished anything at all? And, if so—what? It may not be Sir

Andrew Clark's fault, if he has little work of national importance to point to. It may be that his devotion to his profession has been so complete and so exhausting to his physical and mental energies that there has been no possibility of carrying on at the same time a steady public work which should make for the scientific and practical progress of his age. If that be so, it has at least been, and is, a pity. Men of great powers owe something—nay, they owe much—to a world in which the average man has but small powers: a world in which capable, honest, and noble leadership is the greatest and highest service which any man can render to his fellows. As a leader, as a steady example of what science, when it is associated with the noblest temper and the most fervent "enthusiasm for humanity," can do, Sir Andrew Clark is hardly known. This age of movement, of infinite change, and of infinite possibilities, owes little or nothing to his moulding and fashioning hand.

Mr. Jonathan Hutchinson is also by birth a countryman. Not Scotland, however, but Yorkshire, and we believe the City of York, was the place of his birth. There are some points of resemblance and many of difference between the presidents of the two medical colleges. Sir Andrew Clark is energetic and bustling; Mr. Hutchinson is sober and deliberate. Sir Andrew is strong in work; Mr. Hutchinson can work and wait, too. He has been an "indefatigable notetaker." That is one of the secrets of his fame and success. "An indefatigable notetaker!" What does it mean? An accumulator of facts. But of what use is an accumulation of facts? Of the same use as an accumulation of bricks to an architect. He can build a cottage, a factory, or a palace out of them. Mr. Hutchinson is a scientific architect. He builds surgical cottages, factories, and palaces out of his accumulated facts. The London Hospital gave Sir Andrew Clark his first step to fame. Mr. Hutchinson owes his opportunities to the same hospital. It is a noteworthy fact that these two men, both beginning at the very foot of the professional ladder, should have climbed to such distinguished eminence by means of one and the same hospital, and that hospital placed in one of the poorest and most remote parts of the metropolis. The London Hospital may well be proud of the fame of its distinguished professors.

It may be claimed for the work of Mr. Hutchinson that it has distinctly advanced both the science and art of surgery. His published works mark a clear gain of knowledge, and a philosophic comprehension of diseased processes, rational and experimental methods of treatment, which can hardly be found in equal fullness and completeness elsewhere. As a scientist, and as a surgical practitioner, Mr. Hutchinson has had few equals either in this or any other age. So much we are proud to admit and affirm. But as a citizen of the world, as one who takes a large and deep interest in human affairs outside his specialty, it is unhappily true that Mr. Hutchinson falls far below even Sir Andrew Clark. The present age may justly complain of both these distinguished men, that they are too exclusively devoted to their own special work. For the world at large they seem to have no care. It would be better, even for their own special work, if they would take their places as leaders of at least sections of the community, and help forward thus the scientific knowledge and the practical progress of their less fortunate fellows. The facts of science, and emphatically of medical science; the methods also of science and of the medical art, train the mind for teaching and leading in the wider fields of ordinary life. It is an irreparable loss to the world, it is a loss to medicine itself, it is a grief to all right-minded and far-seeing men, when any powers which might render service to all mankind are exclusively devoted to a strictly limited work. That noblest and most humane of tempers, which characterises alike the highest minds both in religion and science, takes as one of its elementary maxims the doctrine that human capacities are never put to their best use until they are made to serve the whole world in all its highest and worthiest interests. We cannot but deeply deplore the too exclusive devotion of medical men to that limited aspect of their specialty which cares for the sick but almost entirely neglects the well. Medicine can never take rank with religion, science, and law until it puts off the worn-out garments of professional exclusiveness, and clothes itself with the ampler mantle of universal humanity; until it adds to its work of healing the sick, the equally larger and noble duties of seeking the health, the happiness, and the progress of all.

